# Agreements between mean arterial pressure from radial and femoral artery measurements in refractory shock patients

**DOI:** 10.1038/s41598-022-12975-y

**Published:** 2022-05-25

**Authors:** Hemmawan Wisanusattra, Bodin Khwannimit

**Affiliations:** 1 Department of Internal Medicine, Faculty of Medicine, Prince of Songkla University, Hat Yai, Songkhla 90110 Thailand; 2 Division of Critical Care Medicine, Department of Internal Medicine, Faculty of Medicine, Prince of Songkla University, Hat Yai, Songkhla 90110 Thailand

**Keywords:** Medical research, Preclinical research

## Abstract

Radial and femoral artery catheterization is the most common procedure for monitoring patients with shock. However, a disagreement in mean arterial pressure (MAP) between the two sites has been reported. Hence, the aim of this study was to compare the MAP from the radial artery (MAP_radial_) with that of the femoral artery (MAP_femoral_) in patients with refractory shock. A prospective study was conducted in the medical intensive care unit. The radial and femoral were simultaneously measured MAP in the patients every hour, for 24 h. In total, 706 paired data points were obtained from 32 patients. MAP_radial_ strongly correlated with MAP_femoral_ (r = 0.89, p < 0.0001). However, overall MAP_radial_ was significantly lower than MAP_femoral_ 7.6 mmHg. The bias between MAP_radial_ and MAP_femoral_ was − 7.6 mmHg (95% limits of agreement (LOA), − 24.1 to 8.9). In the subgroup of patients with MAP_radial_ < 65 mmHg, MAP_radial_ moderately correlated with MAP_femoral_ (r = 0.63) and the bias was increased to − 13.0 mmHg (95% LOA, − 28.8 to 2.9). There were 414 (58.6%) measurements in which the MAP gradient between the two sites was > 5 mmHg. In conclusion, the radial artery significantly underestimated MAP compared with the femoral artery in patients with refractory shock.

## Introduction

Invasive arterial blood pressure measurement is essential for hemodynamic monitoring in patients with shock admitted to intensive care unit (ICU). Fluid and vasoactive administration are the fundamental management strategies in patients with shock. However, administration of more fluid and large doses of vasopressors are associated with an increased risk of death and organ failure in these shock patients^1,2^. Accurate blood pressure monitoring may lead to proper fluid management and decrease unnecessary vasoactive administration.

Ideally, measurement of central aortic pressures is the gold standard for blood pressure measurement; however, this procedure is invasive and unsuitable for routine clinical practice. Therefore, alternative arteries are used. The most frequently cannulated artery is the radial artery, because of ease of access and fewer complications^3,4^. The second most cannulated artery is the femoral artery. Theoretically, peripheral sites have greater systolic blood pressure (SBP) and lower diastolic blood pressure (DBP) than more central sites, due to the pulse amplification of pressure waves. However, mean arterial pressure (MAP) remains stable, regardless of the site of the arteries^5^.

Nevertheless, the difference in MAP between the radial and femoral arteries has been reported in several critically ill patients, such as cardiac surgery^6,7^, cardiopulmonary bypass^8–10^, liver transplantation^11,12^, and in septic shock patients^13,14^. Previous studies in patients with shock receiving high doses of norepinephrine found that MAP from the femoral artery (MAP_femoral_) was higher than the radial artery (MAP_radial_), ranging from 4.3 to 15 mmHg and 62–75.4% of cases had a MAP gradient ≥ 5 mmHg^13–15^. In contrast some studies in critically ill adult patients^16,17^ and pediatric cardiac surgical patients^18^ reported good agreement between MAP from both sites and concluded that MAP_radial_ was interchangeable with MAP_femoral_ and should be used for blood pressure monitoring in critically ill patients receiving high doses of vasopressors^16,17^.

Thus, definitive information about the gradient between MAP_radial_ and MAP_femoral_, as well as the best choice of blood pressure monitoring in patients with severe shock, peripheral or central arterial catheterization, remains controversial. If the MAP_radial_ underestimates the MAP_femoral_, it may result in an excess of fluid and vasopressor therapies. In addition, the factors associated with the radial-femoral MAP gradient have never been evaluated. The purpose of this study was to determine the correlation and agreement between simultaneous measurements of MAP_radial_ and MAP_femoral_ in patients with refractory shock and to explore the clinical factors associated with the MAP gradient between the two sites.

## Materials and methods

This prospective study was conducted in the medical ICU of a university-affiliated, tertiary referral center in Southern Thailand, from; May 2019 to October 2020. The study was approved by the Human Research Ethics Committee of Faculty of Medicine, Prince of Songkla University (REC: 62-008-14-4) and was registered in the Thai Clinical Trials Registry (TCTR20190603002) and was conducted under the ethical principles of the Declaration of Helsinki. Written informed consent was obtained from the next of kin of all the patients before inclusion to the study.

There is no definite consensus on the definition of refractory shock. However, in general, it can be summarized as; a shock that does not achieve hemodynamic targets, despite the use of high dose vasoactive agents^19,20^. In this study, we defined patients with refractory shock as those receiving an infusion of ≥ 0.5 µg/kg/min of norepinephrine equivalent (1 µg of epinephrine or 100 µg of dopamine, equivalent to 1 µg of norepinephrine)^2,19,20^. The inclusion criteria were as follows: (1) patients with shock who received at least four hours of norepinephrine equivalent ≥ 0.5 µg/kg/min and (2) patients with radial artery catheterization. The exclusion criteria were as follow: (1) contraindication to femoral arterial catheterization, including overlying skin infection, clinical history of severe peripheral vascular disease of the lower limbs, or critical limb ischemia; (2) patients with radial artery catheter malfunctioning, detected by the “fast-flush test” showing overdamping or underdamping of the pressure monitoring systems^4^; (3) use of intra-aortic balloon counterpulsation; and (4) post-cardiac surgery.

### Blood pressure measurements

In our ICU, the radial artery was cannulated with a 20G 3.2-cm catheter (Terumo Surflo, Laguna, Philippines). For femoral catheterization, a 20 cm 16G single lumen catheter (Arrow International, Pennsylvania, USA) was used. The catheters were inserted into the femoral artery using the Seldinger technique under ultrasound-guidance^21^. The arterial catheters were connected to non-compliant pressure tubing and two pressure transducers (TruWave™, Edwards Lifesciences). Both transducers were placed at the same level (phlebostatic axis) and simultaneously zeroed to the atmospheric pressure. The pressure transducer systems were connected to a bedside hemodynamic monitor (Philips Intellivue MP70, Philips Medical Systems, Böeblingen, Germany). A fast-flush test was performed to confirm the adequacy of the frequency response and damping coefficient^4^.

**Figure 1:**
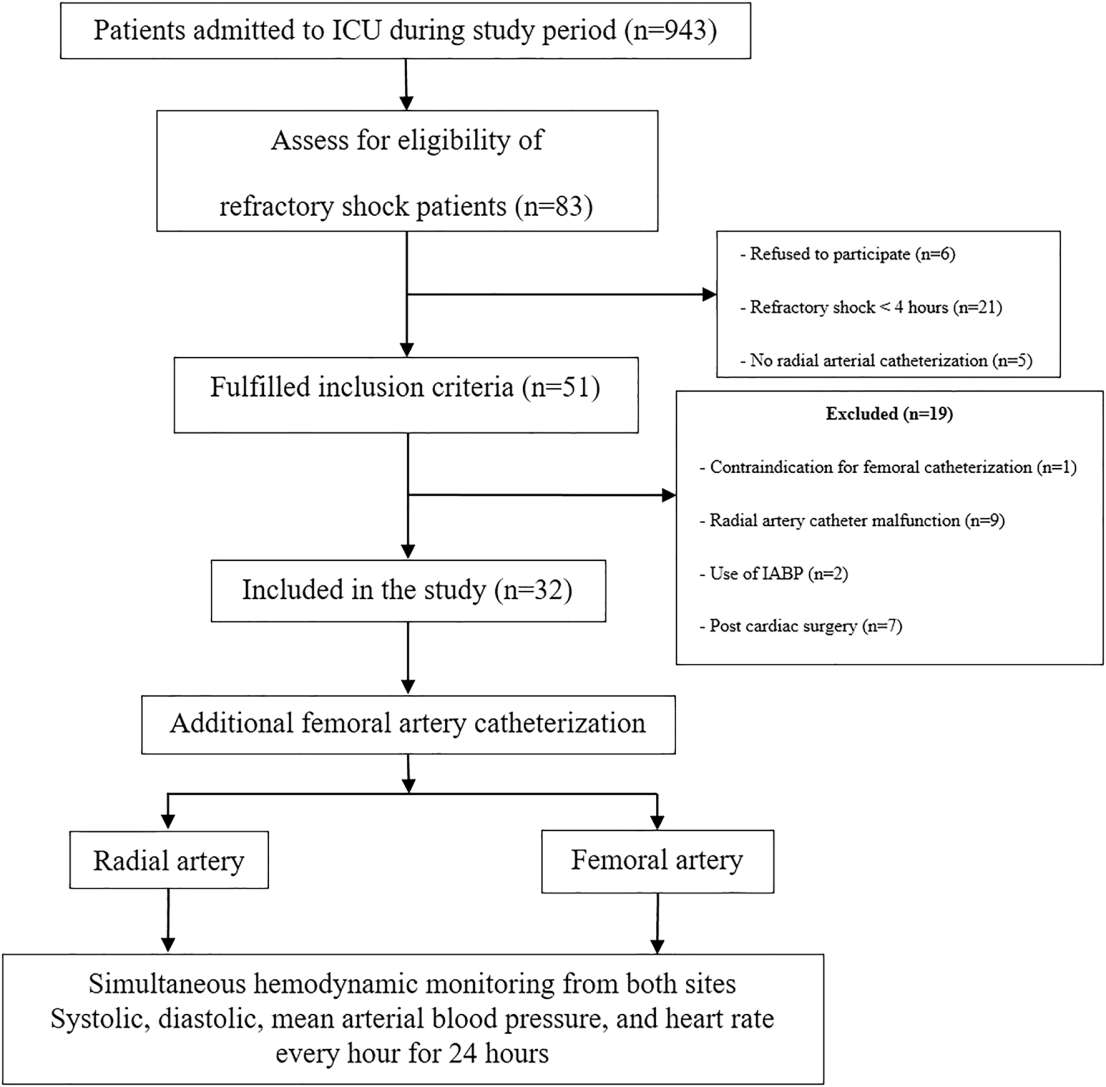
Study flow chart.

The following variables were recorded at inclusion were: age, sex, body weight and, height, Acute Physiology and Chronic Health Evaluation (APACHE) II score, Sequential Organ Failure Assessment (SOFA) score, type of shock, type and dose of vasopressor, and types and sites of infection. ICU mortality was also recorded. SBP, DBP, and MAP from two sites, doses of all the vasopressor provided were recorded after femoral catheterization for 10 min and thereafter, every one hour for 24 h by the ICU nurses (Fig. 1). Complications of arterial catheterization on both sites were observed during ICU stays.

### Statistical analysis

The sample size of the study was calculated by assessing agreement between two methods of measurement by Bland–Altman method^22^. We expected the bias between MAP_radial_ and MAP_femoral_ 4.5 mmHg^14^ and standard deviation of 6 mmHg and allowed maximum difference of 22 mmHg. Therefore, a sample size of 30 patients was calculated (alpha error of 5% and power of 20%).

Categorical variables are expressed as numbers and percentages. The distribution of variables for normality was tested using the Shapiro–Wilk test. Continuous variables are expressed as mean ± standard deviation (SD) or median and interquartile range (IQR) as appropriate.

The Pearson correlation coefficient was used to measure the strength of the linear association between MAP_radial_ and MAP_femoral_. The multilevel mixed-effect model was used to determine the statistical differences between MAP_radial_ and MAP_femoral_^23,24^. The multilevel model was constructed using the arterial site as a fixed effect with random intercepts for both patients and sequential measurement within the patient levels. Bland–Altman analysis with corrected multiple measurements was used to evaluate the agreement between MAP from both sites^25^. The bias and 95% limits of agreement (LOA) of the simultaneous measurements were calculated. Bias was defined as the mean difference between MAP_radial_ and MAP_femoral_. LOAs were calculated as the mean bias ± 2SD. We also performed a bias plot using the Taffé method^26,27^, defining MAP_femoral_ as a standard reference method, and MAP_radial_ as a new method. Briefly, the bias plot shows the scatter plot of the two measurement methods versus the best linear unbiased prediction (BLUP) with the two regression lines added. Taffé uses an empirical Bayes method to compute the BLUP and uses the reference measurements for each subject to estimate their value^26,27^.

The clinically significant differences between MAP_radial_ and MAP_femoral_ was defined as  > 5 mmHg^14,15,17^. The correlation and agreement between MAP from two the sites were assessed in each subgroup of MAP levels and norepinephrine doses. Patients were separated to subgroup of MAP_radial_ measurement < 65 or ≥ 65 mmHg. Regarding, norepinephrine dose group, patients were divided into two groups: those receiving maximum dose of norepinephrine < 1 or ≥ 1 µg/kg/min and those receiving maximum dose of norepinephrine < 0.5 or ≥ 0.5 µg/kg/min. Multilevel mixed-effect logistic regression analysis was used to determine the demographic or hemodynamic factors associated with a significant MAP gradient. Statistical significance was set at p-value < 0.05. All statistical analyses were performed using the Stata 15 software.

## Results

There were 706 paired data obtained from 32 patients, with a mean of 22.1 ± 3.9 data sets per patient. The demographic, clinical characteristics and initial hemodynamic parameters of the patients are shown in Table 1. Septic shock was the most common type of shock in this study (28 patients, 87.5%), and 17 patients (60.7%) had community-acquired infections. Regarding the site of infection, 39.3% were respiratory tract infections, 17.9% were digestive tract and primary bacteremia, and 3.5% were urinary tract and dengue infections. Hemoculture was positive in 14 patients (50%). The most common organisms were *Klebsiella pneumoniae* (27.3%), *Escherichia coli* (18.2%) and *Pseudomonas aeruginosa* (13.6%). All patients required mechanical ventilator support, and ICU mortality rate was 66.7%.

**Table 1:** Characteristics of the study population.

Characteristic	
Age, yr	63.5 [42–72.5]
Male, N (%)	20 (62.5)
Weight, kg	58.2 ± 10.9
Height, cm	162.4 ± 7.8
**Types of shock, N (%)**	
Septic shock	28 (87.5)
Cardiogenic shock	3 (9.4)
Hypovolemic shock	1 (3.1)
APACHE II score	30.1 ± 9.3
SOFA score	13.1 ± 3.7
Norepinephrine (µg/kg/min)	0.65 [0.54–0.88]
Epinephrine (µg/kg/min)	0.33 [0.18–0.54]
Dopamine (µg/kg/min)	7 [3–8]
Norepinephrine equivalent (µg/kg/min)	0.85 [0.7–1.23]
SBP (mmHg)	97.9 ± 26.8
DBP (mmHg)	57.7 ± 10.6
PP (mmHg)	40.2 ± 22.4
MAP (mmHg)	70.7 ± 13.1
HR (mmHg)	123.9 ± 18.3
CVP (mmHg)	15 ± 4

APACHE: acute physiology and chronic health evaluation, CVP: central venous pressure, DBP: diastolic blood pressure, HR: heart rate, MAP: mean arterial pressure, PP: pulse pressure, SBP: systolic blood pressure, SOFA: sequential organ failure assessment.

All the patients received multiple vasoactive agents. Norepinephrine was administered to all patients, 78% and 9.4% of patients received epinephrine and dopamine, respectively. The dosages of each vasopressor are listed in Table 1.

**Figure 2:**
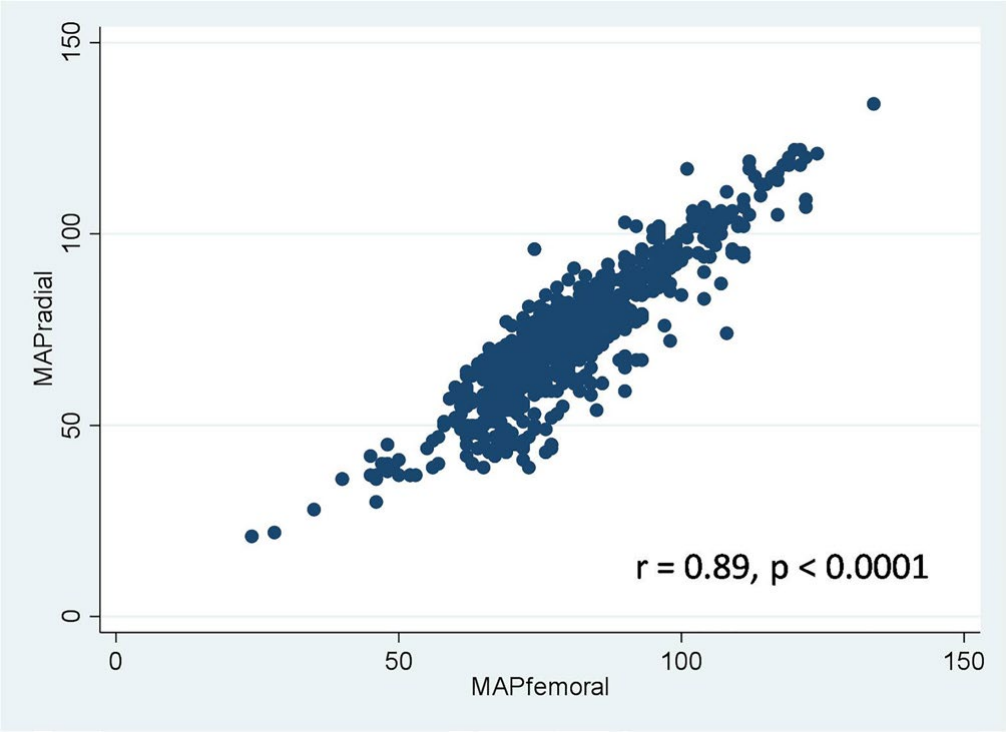
The correlation between mean arterial pressure from the radial (MAP_radial_) and femoral artery (MAP_femoral_).

**Figure 3:**
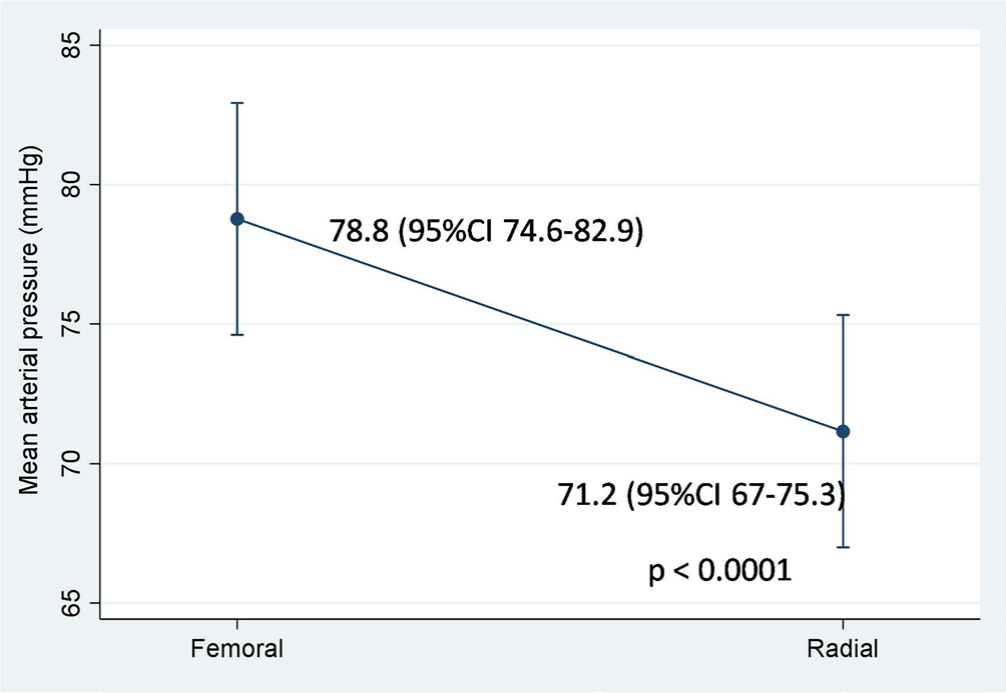
The mean arterial pressure from the radial (MAP_radial_) and femoral artery (MAP_femoral_).

**Figure 4:**
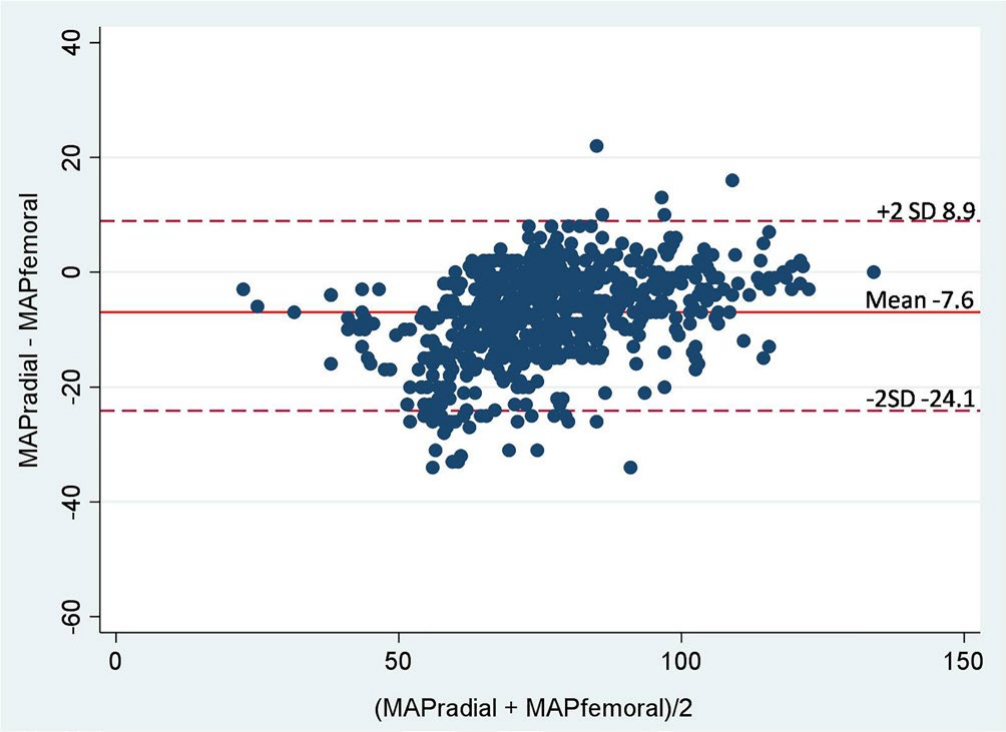
Bland–Altman plot between mean arterial pressure measurement at the radial (MAP_radial_) and femoral artery (MAP_femoral_).

**Figure 5:**
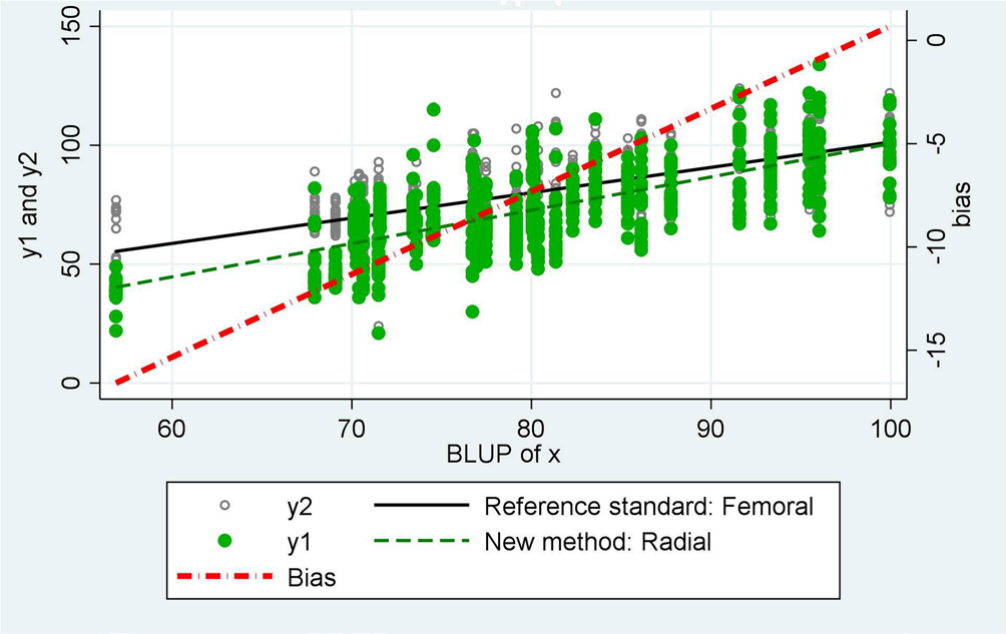
Bias plot between mean arterial pressure measurement at the radial (MAP_radial_) and femoral artery (MAP_femoral_). BLUP: best linear unbiased prediction, X = mean arterial pressure, y1 = mean arterial pressure from radial artery, y2 = mean arterial pressure from femoral artery. The second scale on the right shows the relationship between the estimated amount of bias and the predicted value $\hat{x}_{i}$.

MAP_radial_ strongly correlated with MAP_femoral_ (r = 0.89, p < 0.0001) (Fig. 2). In the multilevel mixed-effect model, the overall mean for MAP_radial_ and MAP_femoral_ were 71.2 (95% CI 67–75.3) and 78.8 (95% CI 74.6–82.9) mmHg, respectively. Thus, MAP_radial_ was significantly lower than MAP_femoral_ by 7.6 mmHg (95% CI 7–8.2) (p < 0.0001) (Fig. 3). The overall mean bias between MAP_radial_ and MAP_femoral_ was − 7.6 mmHg (95% LOA, − 24.1 to 8.9) (Fig. 4). The bias plot, as per the Taffé method, is shown in Fig. 5. The estimated bias (red dash-dot regression line) increased, with a decrease in the level of true MAP (BLUP of the x-axis). When MAP was around 100 mmHg, MAP_femoral_ and MAP_radial_ provided similar values; however, the bias increased (MAP_radial_ progressively lower than MAP_femoral_) when MAP dropped from 100 to 60 mmHg.

Subgroup analysis between patients with MAP_radial_ < 65 mmHg (35.1% of measurements) and those with MAP_radial_ ≥ 65 mmHg was performed. The MAP was markedly discrepant in cases of MAP_radial_ < 65 mmHg; wherein, most of MAP_femoral_ were significantly higher than MAP_radial_. The correlation was fair in group of MAP_radial_ < 65 mmHg (r = 0.63, p < 0.0001) (in the Supplementary file: Fig. S1), with bias of − 13.0 mmHg (95% LOA, − 28.8 to 2.9) (in the Supplementary file: Fig. S2). In contrast, there was also strong correlation if MAP_radial_ ≥ 65 mmHg (r = 0.88, p < 0.0001) (in the Supplementary file: Fig.  S3), with bias of only − 4.2 mmHg (95% LOA, − 17.1 to 8.7) (in the Supplementary file: Fig. S4). The bias in the group of patients with MAP_radial_ < 65 mmHg was significantly higher than that in patients with MAP_radial_ ≥ 65 mmHg (p < 0.0001).

In the subgroup of norepinephrine doses, we found a similar correlation and agreement between MAP_radial_ and MAP_femoral_ in patients receiving norepinephrine lower than 0.5 µg/kg/min and those who received more (Table 2). However, the bias between MAP_radial_ and MAP_femoral_ in the subgroup of patients requiring norepinephrine ≥ 1 ug/kg/min was significantly higher than that in the group of patients requiring norepinephrine < 1 µg/kg/min.

**Table 2:** The correlation and agreement between MAP from radial and femoral arteries in all of the population and each subgroup.

	Correlation (r)	Bias (95% CI)	95% LOA (mmHg)
All (n = 706)	0.89	− 7.6 (− 8.2 to − 7)	− 24.1 to 8.5
MAP ≥ 65 mmHg (n = 458)	0.88	− 4.2 (− 4.8 to − 3.6)	− 17.1 to 8.7
MAP < 65 mmHg (n = 248)	0.63	− 13 (− 13.9 to − 12)	− 28.8 to 2.9
NE < 1 µg/kg/min (n = 485)	0.89	− 6.7 (− 7.4 to − 6)	− 22.3 to 8.9
NE ≥ 1 µg/kg/min (n = 221)	0.87	− 9.6 (− 10.8 to − 8.4)	− 27.3 to 8.1
NE < 0.5 µg/kg/min (n = 170)	0.88	-7.9 (− 9.2 to − 6.6)	− 25.3 to 9.5
NE ≥ 0.5 µg/kg/min (n = 536)	0.89	− 7.5 (− 8.2 to − 6.8)	− 23.7 to 8.6

CI: confident interval, LOA: limit of agreement, MAP: mean arterial pressure, NE: norepinephrine.

There were 414 (58.6%) measurements deviating from the MAP gradient by > 5 mmHg and 235 (33.3%) measurements deviated by > 10 mmHg. The multilevel mixed-effect logistic regression analysis revealed that patients with MAP_radial_ < 65 mmHg (OR 2.34; 95% CI 1.13–4.84, p = 0.02) and body weight (OR 0.89; 95% CI 0.81–0.98, p = 0.01) were associated with significant MAP gradients.

The total complications rate were not statistically different between femoral and radial artery catheterization (25% vs. 12.5%, p = 0.22). Bleeding from the femoral arterial puncture site was observed in 6 (18.7%) patients. All of them were easily controlled and none of these patients needed blood transfusion or further intervention to stop bleeding. Two patients (6.7%) developed non-expand small groin hematoma after femoral catheter removal. In contrast, temporary occlusion at radial artery catheter was observed in 4 (12.5%) patients (detail in the Supplementary file: Table S1).

## Discussion

Invasive blood pressure measurement is important for hemodynamic monitoring and for providing intensive care to patients with shock in the ICU. This prospective study, aimed to investigate the difference between radial and femoral MAP in refractory shock patients receiving high-dose vasopressor therapy. This study found that MAP monitoring at the radial artery significantly underestimated the central arterial pressure as estimated by the femoral artery. Nearly 60% of our refractory shock patients had significant radial-femoral MAP gradients, and MAP_radial_ less than 65 mmHg was an independent risk factor associated with an increased MAP gradient.

Similar to previous studies, this study found disagreement between MAP obtained from the radial and femoral arteries in critically ill patients. Kim et al. demonstrated that the bias between MAP_femoral_ and MAP_radial_ was 4.9 mmHg (95% LOA, − 6.9 to 17) in septic shock patients^14^. Previous study in 24 critically ill patients, in mixed ICU found that bias between femoral and radial MAP was 4.3 mmHg (95% LOA, − 3.4 to 11.9)^15^.

On the other hand, Mignini et al. proposed that measurement of MAP at radial or femoral arteries is clinically interchangeable in critically ill patients. They found that MAP_femoral_ was higher than MAP_radial_ but was not statistically significant, with a mean bias of 3 ± 4 mmHg (95% LOA,16)^16^. A more recent study by Antal et al. also found that MAP_femoral_ and MAP_radial_ had a good correlation and agreement in sepsis patients, with a bias of 1.4 ± 4.7 mmHg (95% LOA, 18.3). The difference between our results and those studies may be related to the different study populations and severity of vasopressors used. Patients with refractory shock in this study were diagnosed with septic shock and required a very high dose of vasopressor (equivalent dose of norepinephrine 0.85 µg/kg/min). However, in the study by Antal et al., only half of their study population was diagnosed with septic shock and they received a lower dose of norepinephrine (0.14 ± 0.17 µg/kg/min) compared to this study.

There were conflicting results regarding the vasopressor dose–effect on the radial-femoral MAP gradients. Previous studies have reported that clinically radial-femoral MAP gradients are commonly observed in patients receiving high doses of norepinephrine administration^13,14^. Kim et al. showed that septic shock patients receiving norepinephrine < 0.1 µg/kg/min had a bias between MAP_femoral_ and MAP_radial_ of 3 mmHg (95% LOA, − 7.2 to 13.1); however, the large discrepancies between MAP were found in patients receiving norepinephrine ≥ 0.1 µg/kg/min, with a bias of up to 6.2 mmHg (95% LOA − 6.0 to 18.3). In contrast, other studies have found that norepinephrine dose was not associated with the MAP gradient. Mignini et al. demonstrated that the bias of MAP_radial_ and MAP_femoral_ was not different between patients receiving high and low dose of vasoactive agents (high vasoactive dose defined as norepinephrine or epinephrine ≥ 0.1 µg/kg/min or dopamine ≥ 10 µg/kg/min). This is consistent with a study in sepsis patients showing that the norepinephrine dose did not the influence the radial-femoral MAP difference^17^. The results of this study support the statement that norepinephrine dose does not influence the radial-femoral MAP gradient.

This study found a high prevalence of clinically significant MAP gradients between the radial and femoral arteries in patients with refractory shock. This is similar to previous studies reporting that 62% of critically ill patients had a MAP difference of at least 5 mmHg and 27–29% of patients had a MAP gradient ≥ 10 mmHg^14,15^. Although, the factors associated with radial-femoral MAP gradients have been extensively investigated, the proper mechanism is still controversial and suggests multifactorial mechanisms for the development of pressure gradients. The radial-femoral MAP gradient may be caused by a decrease in vascular resistance at the level of the hand^28,29^, peripheral vasoconstriction^30^ or a decrease in arterial elastance in the radial artery^8^. Galluccio et al. determined the demographic or hemodynamic factors driving the radial-femoral MAP gradient in critically ill patients. However, they failed to identify any statistically significant associations, including vasopressor dose or any demographic or hemodynamic data^15^.

This study showed that patients with MAP_radial_ < 65 mmHg had a moderate correlation and increased bias between radial and femoral MAP and were also associated with significant MAP gradients between both sites. These results suggest that, in patients with marginally maintained MAP, measured at the radial artery, femoral artery catheterization should be considered for accurate arterial blood pressure monitoring in patients with refractory shock^31^. Monitoring MAP at the femoral artery may avoid future fluid administration or increase of unnecessary vasopressor therapy.

The strength of this study was the use of new statistical analyze to determine bias in the repeated measurement study such as the, bias plot by the Taffé^26^ and the multilevel mixed-effect model^23,24^. However, our study had some limitations. First, most patient with refractory shock in this study had septic shock. Therefore, it may have limited generalizability to other types of shocks. Second, we selected patients with refractory shock who received high doses of vasopressors, which may limit extrapolation for patients with less severe shock. Lastly, the clinical impact of arterial sites on morbidities or mortality was not measure in this study. Therefore, a larger study is required to further investigate the impact of radial and femoral arterial blood pressure monitoring in patients with refractory shock for therapeutic management and patient outcomes.

## Conclusions

The radial artery significantly underestimated MAP when compared with the femoral artery in patients with refractory shock. More than half of the patients had clinically significant MAP gradients. Patients with refractory shock with borderline blood pressure targets, from the radial artery site, should be considered for femoral artery catheterization to obtain accurate measurements of blood pressure.

## Supplementary Information

Supplementary Information.

## Data Availability

The datasets used and/or analyzed during the current study available from the corresponding author on reasonable request.
