# Supplementary material for: Agreements between mean arterial pressure from radial and femoral artery measurements in refractory shock patients
Source: Sci Rep. 2022 May 25;12:8825. doi: 10.1038/s41598-022-12975-y (PMC9133048; doi:10.1038/s41598-022-12975-y)
Supplement: Supplementary file 1 — Supplementary Information. [file 41598_2022_12975_MOESM1_ESM.doc]

Supplementary Materials

**Agreements between mean arterial pressure from radial and femoral artery measurements in refractory shock patients**

**Hemmawan Wisanusattra, Bodin Khwannimit**


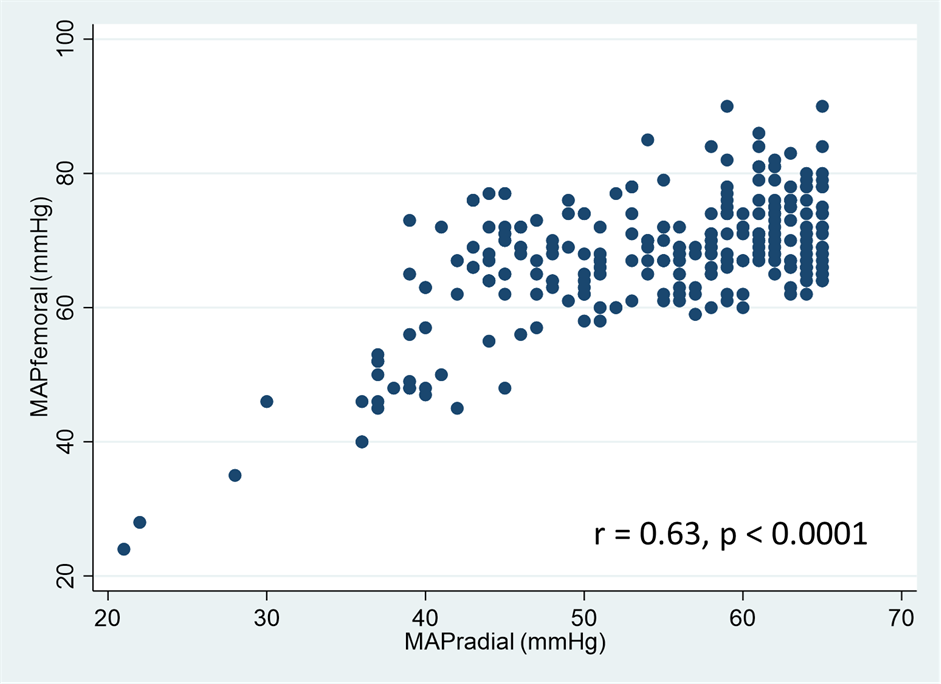


**Figure S1** Correlation of mean arterial pressure from radial (MAP_radial_) and femoral artery (MAP_femoral_) in the subgroup of patients with MAP_radial_ < 65 mmHg.


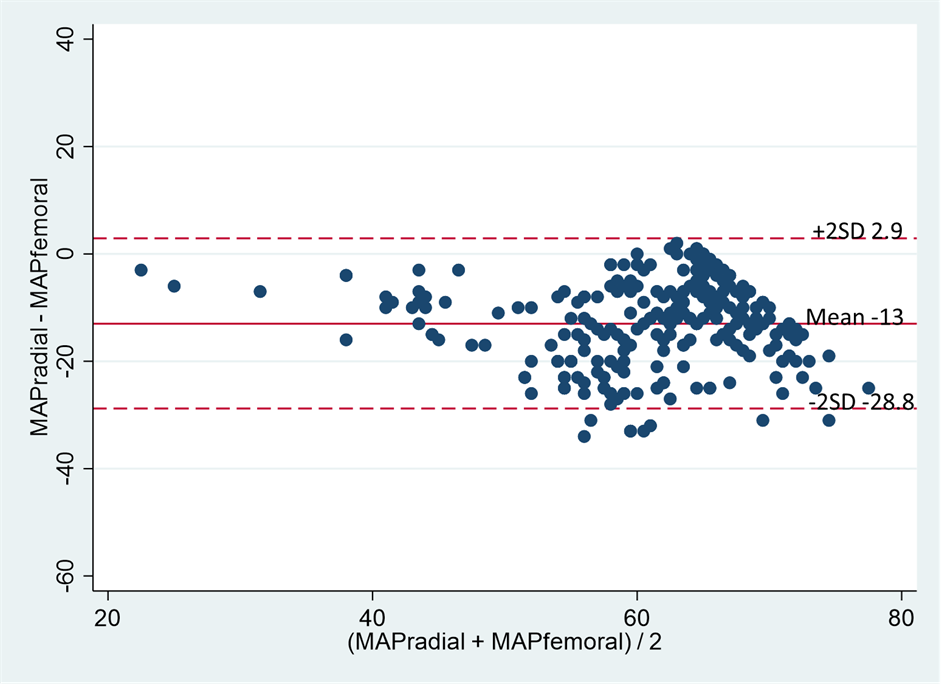


**Figure S2** Bland-Altman plot between mean arterial pressure measurement at radial (MAP_radial_) and femoral artery (MAP_femoral_) in the subgroup of patients with MAP_radial_ < 65 mmHg.

**
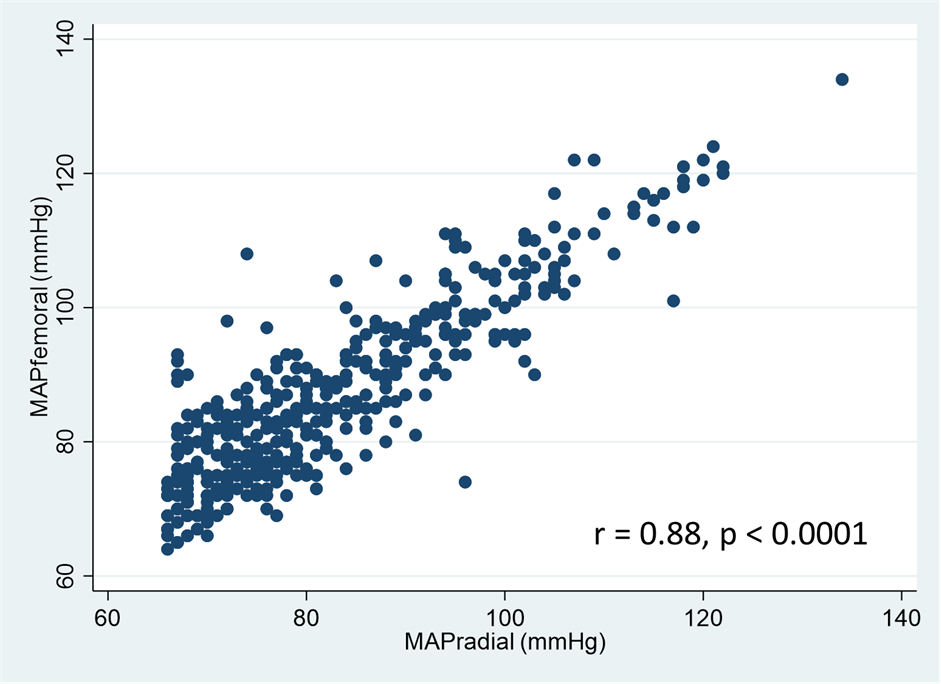
**

**Figure S3** Correlation of mean arterial pressure from radial (MAP_radial_) and femoral artery (MAP_femoral_) in the subgroup of patients with MAP_radial_ ≥ 65 mmHg.

**
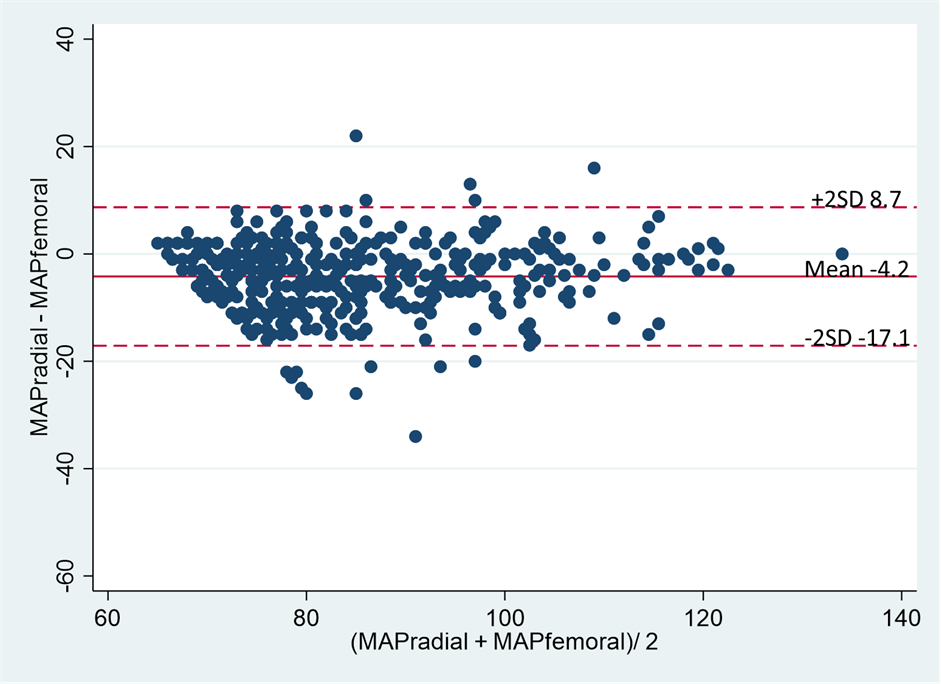
**

**Figure S4** Bland-Altman plot between mean arterial pressure measurement at radial (MAP_radial_) and femoral artery (MAP_femoral_) in the subgroup of patients with MAP_radial_ ≥ 65 mmHg.

**Table S1.** Procedural complications of radial and femoral artery catheterization

| **Procedural complications** | **Radial artery**  **N (%)** | **Femoral artery**  **N (%)** |
| --- | --- | --- |
| None | 28 (87.5) | 24 (75) |
| Hematoma | 0 | 2 (6.7) |
| Bleeding | 0 | 6 (18.7) |
| Temporary occlusion | 4 (12.5) | 0 |
